# Clinical and Bacterial Characteristics of Bloodstream Infections Caused by *Listeria monocytogenes* in Western China

**DOI:** 10.1155/2024/7785327

**Published:** 2024-09-27

**Authors:** Nan Wang, Liuqing Yang, Yu Yuan, Chongyang Wu, Chao He

**Affiliations:** Department of Laboratory Medicine West China Hospital Sichuan University, Chengdu 610041, Sichuan, China

## Abstract

**Objective:**

Bloodstream infections (BSIs) caused by *Listeria monocytogenes* are linked to high mortality of the patients. Case-specific details related to this disease and causative strains in different districts remain to be characterized.

**Methods:**

In this study, medical data of BSIs admitted to West China Hospital from October 2017 to March 2023 were retrieved from the hospital information system. The *in vitro* antimicrobial susceptibility testing and whole-genome sequencing were performed for *L. monocytogenes* strains isolated from blood specimens. The genetic relationship of these strains with those in public databases was also analyzed.

**Result:**

The in-hospital mortality of *L. monocytogenes* BSIs was 25.7% (9/35). The changes in consciousness and elevated serum C-reactive protein (CRP) level were found to be the differential factors of *L. monocytogenes* BSIs (*P* < 0.05). All the 27 strains studied were susceptible to ampicillin, meropenem, and erythromycin. Only 22.2% of them were susceptible to trimethoprim-sulfamethoxazole. The *Listeria* pathogenicity islands 1 (LIPI-1), truncated LIPI-2, and multiple virulence-related genes outside the LIPIs were determined from these strains. Also, 12 sequence types (STs) and 12 clonal complexes (CCs) were identified and classified into clonal lineages I (9/27, 33.3%) and lineages II (18/27, 66.7%), demonstrating genetic differences with the strains in the database. ST451/CC11 (5/27, 18.5%) and ST8/CC8 (4/27, 14.8%) were the common genotypes.

**Conclusions:**

The consciousness change and elevated serum CRP level were found to be the differential factors of *L. monocytogenes* BSIs. Considering the high virulence of the strains, it is needed to pay more attention to the dissemination of the predominant genotype.

## 1. Introduction

*Listeria monocytogenes*, a species of Gram-positive facultative anaerobic bacteria, is capable of causing human listeriosis [1, 2]. If this pathogen penetrates the intestinal barrier, it can access the bloodstream via lymph nodes. Then, it can even penetrate the fetoplacental or blood-brain barrier, resulting in severe and potentially fatal complications, e.g., bloodstream infections (BSIs), premature birth, abortion, or meningitis [1]. The mortality of the patients suffering from *L. monocytogenes* BSIs was reported as high as 46.0% [3]. So, early differential diagnosis of *L. monocytogenes* BSIs is critically important for the treatment of the patients.

The traditional laboratory-based diagnosis of *L. monocytogenes* BSIs relies on the isolation and identification of *L. monocytogenes* from the patient's samples, which usually takes from 3 to 5 days. Moreover, the positivity of blood and cerebrospinal fluid (CSF) culture was reported as only 41.0% and 61.0%, respectively, suggesting a high risk of missed diagnosis [4]. Therefore, early recognition of the characteristics of *L. monocytogenes* BSIs is actually necessary.

Multiple virulence genes were determined from *L. monocytogenes* strains. *Listeria* pathogenicity islands (LIPIs) were reported as the major virulence factors [5–8]. LIPI-1 played an important role in the intracellular parasitic form of *L. monocytogenes* [8]. LIPI-2 was reported to promote the bacteria to internalize into host cells and cross the intestinal, fetoplacental, or blood-brain barriers [5]. LIPI-3 encoded the streptolysin S-like toxin listeriolysin S, which induced cytotoxicity and hemolysis [6]. LIPI-4 encoded a putative phosphotransferase system related to the incidence of neurolisteriosis and maternal-neonatal listeriosis [7] and was most often detected in lineage I [9]. Other virulence-related genes (e.g., *iap*, *clpC*, *clpE*), located outside of the LIPIs, were also described previously [10, 11].

Also, the sequence types (STs) and clonal complexes (CCs) of the strains varied across the countries and regions [7, 9, 12, 13]. The CC1, CC2, CC4, and CC6 were reported in France [7], New Zealand [12], and the Netherlands [13]. CC87 was a predominant type causing invasive listeriosis [9].

Currently, reported cases of *L. monocytogenes* BSIs remain infrequent. Case-specific details related to this disease and causative strains are needed to be characterized. Herein, this study aimed to analyze the characteristics of *L. monocytogenes* BSIs in western China for guiding the prevention and control of this disease.

## 2. Materials and methods

### 2.1. Study Design and Data Collection

**Figure 1:**
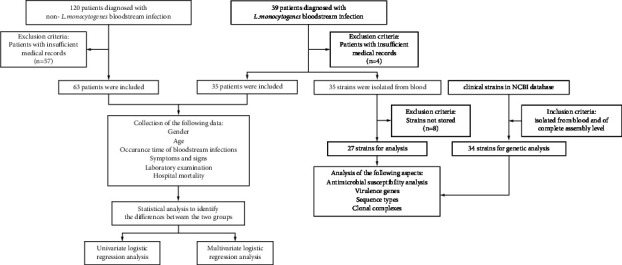
A flowchart of study design and data collection in this study.

A case group of 35 patients suffering from *L. monocytogenes* BSIs admitted from October 2017 to March 2023, as well as a control group of 63 patients diagnosed with BSIs caused by other bacterial species, were enrolled in this study. BSIs were defined as reported previously [14]. The medical data of these patients, such as the demographic data, the clinical signs and symptoms, the findings of laboratory examination, and the data of clinical diagnosis, were retrieved from the hospital information system. A flowchart of the study design and data collection is reported in Figure 1.

### 2.2. Strain Identification and Antimicrobial Susceptibility Testing

The broth of positive blood culture, collected from the patients, was inoculated on the blood agar plate, and then incubated in an incubator (Thermo Fisher, USA) at 37°C with CO_2_. Species identification of the pure colonies on the plates was confirmed by matrix-assisted laser desorption ionization time-of-flight mass spectrometry (Bruker Dalton, Germany), following the 3 steps of sample preparation, mass spectrometry, and data analysis. The *in vitro* susceptibility of the strains to penicillin G (10 unit), ampicillin (10 *μ*g), meropenem (10 *μ*g), trimethoprim-sulfamethoxazole (TMP-SMX) (25 *μ*g), and erythromycin (15 *μ*g) was tested by the disc diffusion or E-test methods, and the results were interpreted according to the guidelines established by the Clinical and Laboratory Standards Institute (CLSI) [15] and European Committee on Antimicrobial Susceptibility Testing (EUCAST) [16].

### 2.3. Whole-Genome Sequencing and Genetic Analysis of *L. monocytogenes* Strains

A total of 27 strains isolated from in-hospital patients were recovered from the storage. Then, bacterial DNA extraction, genomic library preparation, and sequencing were performed using an Illumina HiSeq platform (Illumina, Inc., USA). The results have been deposited at GenBank BioProject no. PRJNA991761. The genes related to virulence were searched in the VFDB database (https://www.mgc.ac.cn/VFs/). MLST was conducted and the BIGSdb-Lm platform (https://bigsdb.pasteur.fr/listeria/) was used for assigning the ST and CC for each strain. Cluster analyses were used to construct a minimum spanning tree generated with PHYLOViZ utilizing the goeBURST algorithm [17]. The phylogenetic relationship and genes heatmap were constructed using the ComplexHeatmap package in R. The data of the blood isolates at the complete assembly level in the National Center for Biotechnology Information (NCBI) database (https://www.ncbi.nlm.nih.gov/) were also incorporated into the genetic analysis.

### 2.4. Statistical Analysis

The data were analyzed using SPSS 27.0 (SPSS Inc., IL, USA). Shapiro–Wilk tests were used to check the normal distribution of continuous variables. Continuous variables that were approximately normally distributed were represented as mean ± sd and compared using *t*-tests. Continuous variables with asymmetrical distributions were represented as median (25th and 75th percentiles) and compared using Wilcoxon tests. Categorical variables were expressed as counts and percentages and compared using chi-square tests. Variables with *P* value < 0.05 were candidate variables for the multivariable logistic regression analysis.

## 3. Results

### 3.1. Clinical Characteristics of *L. monocytogenes* BSIs

**Figure 2:**
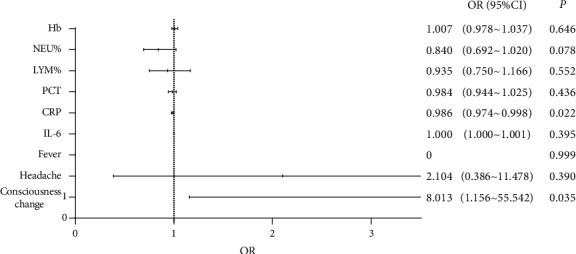
Multivariate logistic regression analysis of the differential factors for *Listeria monocytogenes* bloodstream infections in this study. Hb: hemoglobin; NEU%: neutrophilic granulocyte percentage; LYM%: lymphocyte percentage; PCT: procalcitonin; CRP: C-reactive protein; IL-6: interleukin 6; OR: odds ratio; CI: confidence interval. Error bars indicate 95% CI.

The characteristics of the case group with *L. monocytogenes* BSIs and the control group with BSIs caused by other bacterial species were analyzed. As shown in Table 1, the clinical symptoms or signs and inflammatory indicators were found to be statistically different between the two groups (*P* < 0.05). By multivariate logistic regression analysis, the consciousness change (*P*=0.035) and elevated serum CRP level (*P*=0.022) were found to be the differential factors for *L. monocytogenes* BSIs (Figure 2).

### 3.2. *In Vitro* Susceptibility of *L. monocytogenes* Strains

As described in Table 2, all the 27 strains tested were susceptible to ampicillin, meropenem, and erythromycin. However, 3.7% of them were resistant to penicillin G and 77.8% of them were resistant to TMP-SMX.

### 3.3. Virulence-Related Genes and Genotypes of *L. monocytogenes* Strains

The virulence-related genes, including LIPI-1, truncated LIPI-2, *iap/cwhA*, *clpC*, *clpE*, *oatA*, *lap*, *lapB*, *bsh*, *oppA*, *hbp2*, *visS* and *virR*, were detected from the strains studied (Table S1). By MLST analysis, 12 STs and 12 CCs were assigned for these strains. ST451/CC11 (5/27, 18.5%) and ST8/CC8 (4/27, 14.8%) were frequently identified.

### 3.4. Genetic Relationships of *L. monocytogenes* Strains in This Study with Those in the Public Databases

**Figure 3:**
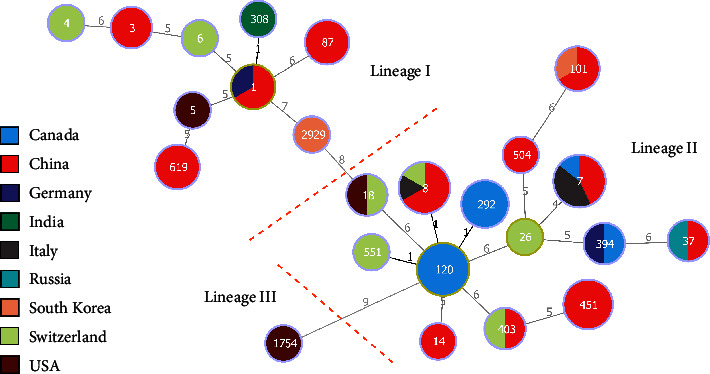
Minimum spanning tree analysis based on multilocus sequence typing data of 27 strains of *Listeria monocytogenes* isolated from blood culture in this study and 34 ones in the NCBI database. Sequence type (ST) numbers are shown in nodes, while the number of allelic differences between nodes is represented on branches. Different colors of the nodes represent different origin countries. Lineages I, II, and III are separated by two orange dotted lines.

**Figure 4:**
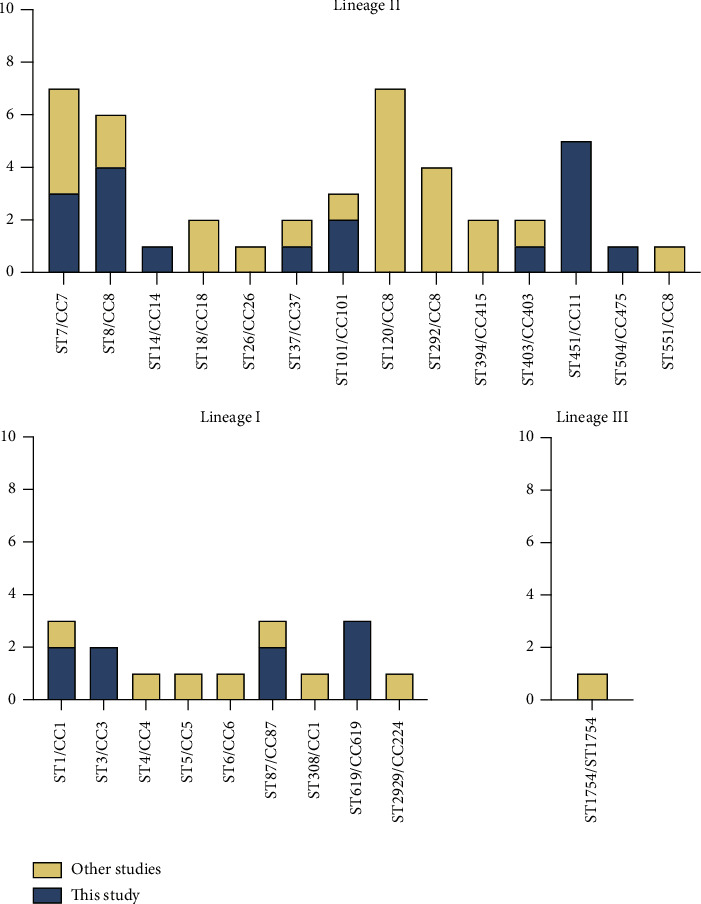
Genotypes identified for 27 strains of *Listeria monocytogenes* isolated from the blood culture in this study and 34 ones in the NCBI database. STs: sequence types; CCs: clonal complexes.

**Figure 5:**
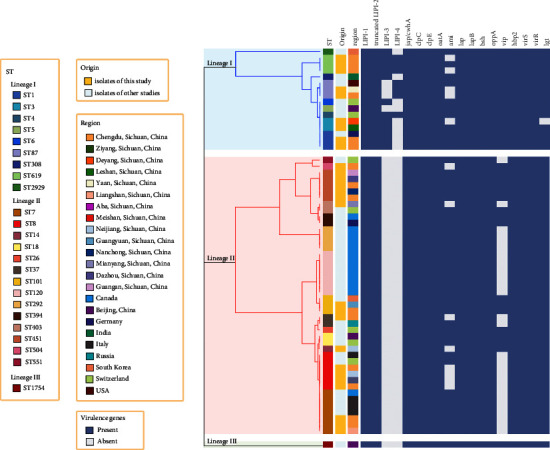
Phylogenetic relationship and gene heatmap of 27 strains of *Listeria monocytogenes* isolated from blood culture in this study and 34 ones in the NCBI database. The profiles of multilocus sequence typing were used to construct a clustering tree with branches colored according to phylogenetic lineages (lineage I: blue; lineage II: red; lineage III: green). The presence of virulence-related genes was marked in blue.

Up to July 10th, 2023, 34 strains of complete assembly level from blood were retrieved from NCBI database. For 61 strains, isolated from the blood of the patients, including 27 strains in our study and 34 strains in the NCBI database, a total of 24 STs and 20 CCs were determined, as shown in Figure 3. The distribution of STs and clonal lineages of all these 61 strains was described in Figure 4. The strains in our study were classified into lineage I (9/27, 33.3%) and lineage II (18/27, 66.7%). In addition, 3.0% (1/34) of the strains in the NCBI database were classified into lineage III. The phylogenetic relationship and gene heatmap of all these 61 strains are demonstrated in Figure 5.

## 4. Discussion

*L. monocytogenes* is the most common species of *Listeria* spp. which affects humans [2]. We found that consciousness change and elevated serum CRP level were the differential factors for *L. monocytogenes* BSIs. In previous studies, above 90.0% of patients with *L. monocytogenes* BSIs had elevated serum CRP, while 69.0% of cases had elevated serum procalcitonin (PCT) [3]. Approximately 30.0% of the cases developed meningitis in other studies [3, 18] but this ratio was lower (25.7%) in our study. The in-hospital mortality of the patients with *L. monocytogenes* BSIs in this study was 25.7%, lower than 35.2% in the other study [3].

The *L.monocytogenes* strains in this study showed high *in vitro* susceptibility to the antibiotics with the exception of TMP-SMX, consistent with prior reports from Belgium [19], France [20], and Italy [21]. By contrast, the strains from other areas of China from 2013 to 2019 exhibited a lower resistance rate to TMP-SMX (3/360, 1.0%) [9]. Given that TMP-SMX is frequently used as an alternative treatment, this finding suggested that it should not be favored as an empirical treatment in western China.

As shown in Table S1, all the *L. monocytogenes* isolates in this study harbored LIPI-1 and truncated LIPI-2. However, LIPI-3 was detected in the 7 isolates (25.9%) of lineage I, in line with a prior report that LIPI-3 was only present in a subset of lineage I [6]. LIPI-4 was detected in two ST87/CC87 isolates and three ST619/CC619 isolates, and was also previously reported in CC4, ST87, and ST619 isolates [7, 22, 23]. The wide presence of LIPIs and virulence genes located outside of LIPIs suggests the high virulence of the isolates. The high virulence of the strains might lead to unfavorable clinical outcomes of *L. monocytogenes* BSIs, as demonstrated in the other reports [7, 9, 23].

The genetic relationship analysis of the 27 strains isolated from blood culture in this study and 34 of those in the NCBI database, as shown in Figures 3 and 5, indicated that the genetic links among these strains were weak, as suggested in other districts [24]. The predominant genotypes in local areas should be paid more attention to.

In conclusion, the consciousness change and elevated CRP level were found to be the differential factors of *L. monocytogenes* BSIs in this study. The strains in western China showed high *in vitro* susceptibility to antibiotics but carried multiple virulence genes. It is needed to pay more attention to the dissemination of the predominant genotype.

## Figures and Tables

**Table 1:** Clinical characteristics of bloodstream infections included in this study.

Characteristics	Case group (*n* = 35)	Control group (*n* = 63)	*P*
Male [*n*, (%)]	20 (57.1)	43 (68.3)	0.271
Age (*x* ± s, years)	57.2 ± 15.9	53.2 ± 13.4	0.185
Symptoms and sighs [*n*, (%)]			
Fever	28 (80.0)	61 (96.8)	0.016
Gastrointestinal symptom	19 (54.3)	29 (46.0)	0.434
Consciousness change	15 (42.9)	13 (20.6)	0.020
Headache	16 (45.7)	10 (15.9)	0.001
Laboratory examination of blood specimens at admission			
Hb (*x* ± s, g/L)	110.1 ± 29.2	95.9 ± 27.0	0.017
PLT [M (P25, P75), ×10^9^/L]	137.0 (93.0, 203.0)	113.0 (70.0, 175.0)	0.178
WBC [M (P25, P75), ×10^9^/L]	11.6 (5.7, 17.0)	9.9 (7.2, 13.9)	0.767
NEU% [M (P25, P75)]	83.6 (77.2, 87.0)	87.6 (82.9, 91.2)	0.002
LYM% [M (P25, P75)]	8.3 (4.9, 15.3)	5.5 (4.0, 8.3)	0.004
MONO% [M (P25, P75)]	6.9 (5.5, 8.8)	5.6 (3.6, 8.5)	0.078
AST [M (P25, P75), U/L]	31.0 (19.0, 62.0)	32.5 (21.8, 66.0)	0.749
ALT [M (P25, P75), U/L]	31.4 (15.0, 55.0)	25.5 (14.8, 55.5)	0.406
LDH [M (P25, P75), U/L]	295.5 (218.8, 424.3)	242.5 (191.8, 368.3)	0.246
Na [M (P25, P75), mmol/L]	133.3 (130.0, 137.0)	134.3 (130.7, 138.3)	0.390
Glu [M (P25, P75), mmol/L]	7.5 (5.8, 9.8)	7.7 (6.2, 10.9)	0.530
PCT [M (P25, P75), ng/mL]	0.5 (0.2, 2.3)	3.7 (1.2, 23.9)	<0.001
CRP [M (P25, P75), mg/L]	37.4 (21.3, 101.2)	146.0 (92.7, 184.3)	<0.001
IL-6 [M (P25, P75), pg/mL]	50.6 (23.0, 126.9)	205.1 (78.8, 534.4)	<0.001
In-hospital mortality [*n*, (%)]	9 (25.7)	12 (19.0)	0.441

Case group, 35 patients suffering from *L. monocytogenes* BSIs; control group, 63 patients with BSIs caused by the other bacterial species; Hb, hemoglobin; PLT, platelet; WBC, white blood cell; NEU%, neutrophilic granulocyte percentage; LYM%, lymphocyte percentage; MONO%, monocyte percentage; AST, aspartate aminotransferase; ALT, alanine aminotransferase; LDH, lactic dehydrogenase; Na, sodium; Glu, glucose; PCT, procalcitonin; CRP, C-reactive protein; IL-6, interleukin 6; M, median; P25, lower quartile; P75, upper quartile.

**Table 2:** *In vitro* susceptibility of *Listeria monocytogenes* strains isolated from blood culture in this study.

Antibiotics	Resistance (*n*, %)	Susceptibility (*n*, %)	MIC (*μ*g/mL)
Penicillin G	1 (3.7%)	26 (96.3%)	0.002–0.5
Ampicillin	—	27 (100.0%)	0.032–0.5
Meropenem	—	27 (100.0%)	0.048–0.125
TMP-SMX	21 (77.8%)	6 (22.2%)	—
Erythromycin	—	27 (100.0%)	0.125–0.5

TMP-SMX, trimethoprim-sulfamethoxazole. The susceptibility to TMP-SMX was tested by disc diffusion.

## Data Availability

The sequencing data of the strains analyzed in this study can be accessed in the NCBI database (https://www.ncbi.nlm.nih.gov/) at GenBank under the BioProject no. PRJNA991761.

## Supplementary Materials

Supplementary Materials The details of the virulence-related genes and genotypes of the *Listeria monocytogenes* strains isolated from blood culture in this study are described in Supplementary Table S1.
