# Supplementary material for: Clinical and Bacterial Characteristics of Bloodstream Infections Caused by Listeria monocytogenes in Western China
Source: Can J Infect Dis Med Microbiol. 2024 Sep 27;2024:7785327. doi: 10.1155/2024/7785327 (PMC11452242; doi:10.1155/2024/7785327)
Supplement: Supplementary Materials — The details of the virulence-related genes and genotypes of the Listeria monocytogenes strains isolated from blood culture in this study are described in Supplementary Table S1. [file 7785327.f1.docx]

**Table S1 Virulence-related genes and genotypes of *Listeria monocytogenes* strains isolated from blood culture in this study**

| Strains isolated | Virulence genes | | Genotypes | |
| --- | --- | --- | --- | --- |
|  | LIPIs (n) | genes outside the LIPIs (n) | lineage I (n) | lineage II (n) |
| Blood  (n=27) | LIPI-1+LIPI-2 (18),  LIPI-1+LIPI-2+LIPI-3 (4),  LIPI-1+LIPI-2+LIPI-4 (2),  LIPI-1+LIPI-2+LIPI-3+LIPI-4 (3) | *iap/cwhA* (27), *clpC* (27),  *clpE* (27), *oatA* (27),  *ami* (14), *lap* (27),  *lapB* (27), *bsh* (27),  *oppA* (27), *vip* (18),  *hbp2* (27), *virS* (27),  *virR* (27), *lgt* (26) | ST1/CC1 (2),  ST3/CC3 (2),  ST87/CC87 (2),  ST619/CC619 (3) | ST7/CC7 (3),  ST8/CC8 (4),  ST14/CC14 (1),  ST37/CC37 (1),  ST101/CC101 (2),  ST403/CC403 (1),  ST451/CC11(5),  ST504/CC475 (1) |

Note: LIPIs, Listeria pathogenicity island; STs, sequence types; CCs, clonal complexes
